# Sex difference in body image, exercise motivation and social comparison among Instagram users: a cross sectional study

**DOI:** 10.12688/f1000research.134799.2

**Published:** 2024-06-14

**Authors:** Aysha Nimiya, Vasudha K.G, Sharanya B Shetty, Keshava Pai, Reshma N S, Radhika K, Mariella D'Souza, Priyanka D'Souza

**Affiliations:** 1 Department of Psychiatry, Kasturba Medical College, Mangalore, Manipal Academy of Higher Education, Manipal, India

**Keywords:** Instagram, Body Image, Exercise Motivation, Social Comparison

## Abstract

In the 21 ^st^ century, impact of social media, particularly Social Networking Sites (SNSs) has been linked to a wide range of human beliefs and expectations. Growing body of research has indicated that body image concerns along with exercise motivation and social comparison are on the rise among young adults. The present study aimed to examine the sex difference in body image, exercise motivation and social comparison among people who use Instagram in the age group 20-30. A total of 212 participants (men=106, women=106) aged 20-30 years, who are users of Instagram completed Body Self Image Questionnaire Short Form as a measure of Body image, Exercise Motivation Inventory – 2 as a measure of Exercise Motivation and Instagram as a Tool for Social Comparison as a measure of Social Comparison. Results showed that a significant difference in body image exist across gender with body image issues higher among females and significant difference in exercise motivation across gender with exercise motivation higher among males. No sex differences were seen in social comparison. It was concluded that body image concerns are higher among females and the drive for exercise is higher among males who used Instagram. It was found that body image concerns were higher among people who exercised regularly as well as among those who followed fitness related pages on Instagram as compared to those who did not. These results provide an insight into the sex differences between the variables and future directions can be aimed at conducting an in-depth analysis using body image, exercise motivation and social comparison.

## Introduction

In this era of developing technology, many studies have been focusing on how social media and social networking sites (SNSs) relate to body image issues. Research indicates that peer contacts, the prevalence of sharing pictures, and the availability of cell phone automation all contribute to the possibility that people who use SNSs may internalize the “skinny” prototype and self-embody ( Lewallen, Behm-Morawitz, 2016). The role of exercise in understanding body image distortions are undeniable in the contemporary world ( Littrell, 2017). The harmful possible side effects of engagements in physical activities to enhance one’s body image has been intensified by the SNSs, especially *via* applications like Facebook, Instagram, Snapchat etc. Research has consistently shown that celebrities, fashion influencers, peer and media effects are particularly important in influencing social comparison among both men and women. Thus, the statistics imply that social comparisons of physical appearance and the internalization of notions of beauty remain more prominent in people who actively use social networking ( Lewallen, Behm-Morawitz, 2016; Brown & Tiggemann, 2016).

Social media use refers to use of various kinds of artificial intelligence, such as social networking platforms and personal websites or online columns, through which users build online communities to share knowledge, concepts, beliefs, exclusive and intimate messages, and other contents such as photos & videos ( Cohen, Newton-John & Slater, 2017). According to recent data, around 59% world’s population use social media, which in number approximates to 4.76 billion and about 137 million people have created online profiles/accounts within the last 12 months with an average daily time of 2h and 31 m spent in these social networking websites ( Kemp S. Digital, 2023: Global Overview Report [Internet], 2023) The various motives for engaging in social media includes entertainment (25.51%),personal utility (12.34%), information seeking (7.53%), convenience (6.21%) and altruism (4.90%) ( Al-Menayes, 2015). Recent studies have looked at how social media and social networking sites (SNS) are connected to body image issues ( Eckler, Kalyango & Paasch, 2017). Instagram is a distinctive social network application which allows users to connect with friends and post photos and videos ( Panjrath & Tiwari, 2021). As of October 2021, the number of Instagram users have boosted up to 1.38 billion. While 70% of Instagram users are under 35 years, the proportion of users over 35years has grown every year since 2018, similar to Facebook ( Statista Research Department, 2022). The terms “body image disturbance,” “body dissatisfaction,” or “body image concern” are frequently used in the literature to describe an unfavorable, displeased impression of one’s body ( Fardouly *et al*., 2015). In Western nations, both women and men accept the “thin ideal” conception of women portrayed by media and society. Women oftentimes feel a gap between their existing real bodies and the elusive ideal female body, which eventually paves the way for body dissatisfaction ( Mills, Musto, Williams, & Tiggemann, 2018). However, in the past few decades, the concept of ‘perfect’ male body has gained popularity. According to research, media representations of the ideal man have evolved through time and are now highly exaggerated and promoted to men through publications and television ( Singh, Parsekar, & Bhumika, 2016).

Exercise motivation refers to individuals’ reasons for exercising (participation motives) in determining long-term adherence to regular physical activity ( Markland & Hardy, 1993). Exercise motivation is primarily predicted by the perception of one’s physical appearance as being favourable or unfavourable. One of the specific reasons people exercise is to preserve or improve their desired physical appearance. Weight management, looks, and body dissatisfaction have all repeatedly ranked highly as factors that motivate people to exercise, demonstrating the connection between body image and exercise participation ( Halder & Mondal, 2020). Even though studies on body image with respect to social media usage have primarily focused on women, it has been discovered that male millennials are increasingly using SNSs such as Instagram and fitness hashtags to communicate and gain knowledge about fit bodies, raising the likelihood of body image concerns.

Lean and toned bodies are frequently portrayed in media, and diet and exercise are frequently promoted for their aesthetic rather than health benefits ( Holland & Tiggemann, 2016). This evidence points to the fact that people are inclined to desire to change the dimensions of their bodies resulting from the media and societal influence to keep up with the idealized convention of a thin and toned body for women and a lean and muscular physique for men, with a desire for the rewards for looking good, and the health benefits of maintaining the prescribed body weight ( Gültzow, Guidry & Schneider, 2020).

Another crucial element that has been identified to be a key component in understanding body image is social comparison. It refers to the cognitive judgments that people make about their own attributes compared to others ( Yang, 2016). According to Festinger’s social comparison theory (1954), people frequently compare their lives and selves to those of others based on the information they learn about others ( Hobza *et al.*, 2007). Social comparison comes in two main forms. Comparison to those we believe to be less fortunate than ourselves in some way, or downward social comparison, tends to improve happiness and self-worth. Upward social comparison or comparing oneself to someone we believe to be socially superior to oneself, typically results in a bad mood and can jeopardise one’s ability to evaluate oneself. When there are differences between oneself and the comparison standard, people are motivated to modify themselves in order to resemble the comparison standard more closely, which serves to promote the self ( Vogel, Rose & Okdie, 2015; Yang, 2016). There is evidence that various SNS information, including user profiles, “likes,” and comments, can cause social comparison ( de Vries *et al*., 2018).

## Current study

The media and popular culture endorse a thin beauty standard for women, which women and men in Western cultures simply accept. However, in the past few decades, the concept of ‘perfect’ male body has gained popularity. As a result, men, these days are also forced to work towards building an ideal body that is challenging to achieve ( Chatzopoulou, Filieri & Dogruyol, 2020). In line with these findings, research has shown that men, like women, feel discontented and distressed when they encounter same sex individuals with an ‘ideal body’ ( Prichard *et al*., 2020). Although this preference towards bodily stimuli is well-documented in female populations, research on it in male populations is very scarce ( Daniel Talbot & Daniella Saleme, 2022). However, the existence of enormous literature in the western countries on body image and exercise motivation across gender is not necessarily globally representative, stemming from the finite number of investigations conducted in the Indian context. Additionally, there are very few published studies on the role of social comparison in body image of males, because of which Sex differences in social comparison have not been deeply analyzed. With the existing literature, there is insufficient research on use of social media, body image, exercise motivation and social comparison in men as most of the studies have been focused on women. For instance a study titled “Attractive celebrity and peer images on Instagram: Effect on women’s mood and body image”( Brown & Tiggemann, 2016), another study titled “Facebook Use and Negative Body Image among U.S. College Women” ( Eckler, Kalyango & Paasch, 2017) and similarly, another study also focused on women titled, “Why Them, Not Me?”: A Study Exploring The Impact Of Following Fashion Influencers on Instagram on Body Image Satisfaction of Adolescent Girls and Middle-aged Women ( Panjrath & Tiwari, 2021). These are few among the studies that mostly focused on women and their body image disturbances. So, we believe a comparative study is required to throw light on how these variables operate in women as well as in men. We aimed to study the Sex difference in body image, exercise motivation and social comparison among people who use Instagram in the age group 20-30 years.

## Methods

### Participants and study setting

A cross-sectional study was conducted in India, where participants were recruited through various online platforms. Two Google forms were created, in which the first one consisted of the participant study information sheet, inclusion criteria and informed consent. The second one consisted of the three questionnaires used in the study. The first Google form was circulated through various online platforms such as Whatsapp, Instagram and Gmail. After a period of 15 days, a reminder was sent to the participants regarding the same. Participants who met the inclusion criteria of the study that is belonging to the age group 20-30 and using an Instagram account, participants who identified themselves as male or female and who gave consent to the study were forwarded the second Google form consisting of the questionnaires. The data was collected through convenience sampling between August 2022 to December 2022. The predictor variable in the study is Instagram use and outcome variables in the study are body image, exercise motivation and social comparison. This study is based on the ‘The Strengthening of Reporting Observational Studies in Epidemiology (STROBE) statement guidelines ( Elm *et al*., 2007). The study flow as per STROBE guidelines is depicted in the extended data ( Vasudha & Nimiya, 2023).

A sample size of 212 was calculated, considering a confidence level of 95%, with margin of error of 10%. Thus, 212 participants (males = 106, females = 106) aged between 20 and 30 years with an active Instagram account (Male; M = 22.76, SD = 1.84), (Female; M = 23.05, SD = 2.21) were recruited from different states across India to participate in the study. Participants without an active Instagram account as well as those with a history of physical/mental illness were excluded from the study. This computation was used to maintain a balance between statistical power and feasibility. The sample size was judged suitable for detecting moderate effect sizes in our comparisons. The selected sample size was sufficient for preliminary insights into sex differences in body image, exercise motivation, and social comparison among Instagram users aged 20-30 in India. We ensured that there was an equal representation of male and female participants (106 men and 106 women) to reduce the possibility of bias and increase the robustness of our results. Participants were recruited *via* various social media platforms to take part in the study. Participation was voluntary. This age range was chosen not only for convenience, but because individuals of the prescribed age range are the heaviest users of Instagram ( Kemp S. Digital 2023: Global Overview Report [Internet], 2023).

### Design

The research design employed in the study was quantitative comparative cross-sectional design which includes the analysis of data of variables collected at one given point in time across a sample population of a pre-defined subset. This method was applied to investigate the Sex difference in Instagram use with regards to body image, exercise motivation and social comparison.

### Materials

*Demographics*

Participants were asked to report demographic details such as age, sex, educational qualification, history of mental/physical illness, Instagram activity status, duration of Instagram use, number of celebrities followed on Instagram, number of fitness related pages followed on Instagram, frequency of posting exercise related pictures, exercise activity status and exercise routine: (Place of exercise, Duration of exercise, exercise with/without instructor).

*Body Self Image Questionnaire Short Form (BSIQ SF)*

The Body Self Image Questionnaire (BSIQ) is a 27-item measure developed by Rowe (2005) comprising of nine, three-item scales assessing the person’s thoughts and feelings about the human body which are: Overall Appearance Evaluation; Health Fitness Influence; Investment in Ideals; Health-Fitness Evaluation; Attention to Grooming; Height Dissatisfaction; Fatness Evaluation; Negative Affect and Social Dependence. Before using the scale in our study we obtained permission from the author to use the scale in this study. Respondents indicate the degree to which they think each item is true regarding their body using a five-point Likert scale, with 1 = Not at all True of Myself, 2 = Slightly True of Myself, 3 = About Halfway True of Myself, 4 = Mostly True of Myself, and 5 = Completely True of Myself. With 5 being the lowest possible score and 135 being the maximum score, the higher the score, the greater the body image concerns. All nine sub-scales show adequate internal consistencies, as demonstrated by Cronbach alphas ranging from 0.68 to 0.92 ( Rowe, 2005).

*Exercise Motivation Inventory-2 (EMI-2)*

The Exercise Motivations Inventory-2 (EMI-2) is a 51-item scale developed by Markland and Ingledew (1997) comprising of nine four-item scales and five three-item scales assessing individuals’ reasons for exercising (participation motives) in determining long-term adherence to regular physical activity which are Stress Management, Revitalization, Enjoyment, Challenge, Social Recognition, Affiliation, Competition, Health Pressures, Ill- Health Avoidance, Positive Health, Weight Management, Appearance, Strength & Endurance, Nimbleness. Respondents indicate, by circling the appropriate number, whether or not each item is true for them personally or would be true for them personally if they did exercise. If the statement is considered to be not at all true, ‘0’is circled. If the statement is very true indeed, ‘5’ is circled. If the statement is partly true, then ‘1’, ‘2’, ‘3’ or ‘4’ are circled, according to how strongly the person feels that it reflects why he/she exercise or might exercise. With 5 being the lowest possible score and 255 being the maximum score, higher the score, greater the exercise motivation. All fourteen sub-scales show adequate internal consistencies, as demonstrated by Cronbach alphas ranging from 0.69 to 0.92 ( Markland & Hardy, 1993).

*Instagram as a tool for Social Comparison*

Instagram as a tool for Social Comparison is a 10-item measure which was developed by the Principal Investigator for assessing how individuals compare themselves with celebrities, peers or others with respect to their Instagram usage (see extended data). This includes aspects such as individuals’ feeling and thoughts towards posts/likes/comments/shares of fitness related pictures and videos in Instagram. Respondents indicated what extent to which they think the statements are true for them with each item using a five-point Likert scale, with a = Strongly Disagree, b = Disagree, c = Neutral, d = Agree and e = Strongly Agree. Some of the sample questions are as follows: “I take into consideration the views/likes/comments/shares I receive for my posts/stories”, “I judge myself based on the number of followers and likes”, “I repeatedly change my Instagram profile picture”, “I always think about what others might be thinking about the pictures/videos that I post in my feeds/stories”. With 5 being the lowest possible score and 50 being the maximum score; the higher the score, greater the magnitude of social comparison. The questionnaire was validated by three experts from the field. The name of the scale was changed from ‘Social Comparison and Instagram Use scale’ to ‘Instagram as a tool for Social Comparison’ as per experts’ suggestion. Certain other modifications were suggested, and these suggestions were taken into consideration and the modified questionnaire was used for data collection ( Bessenoff, 2006).

### Procedure

The author’s Institutional Ethics Committee approved this study (Protocol No: IEC KMC MLR 04-2022/127). The population of this study were males and females in the age group 20-30 residing in different states across India. The independent and dependent variables in the study are Instagram usage and Body image, Exercise motivation, Social Comparison respectively. Participants gave written, informed consent before the commencement of the study.

The study was conducted in two phases:

*Pilot phase -* The pilot study was conducted from June 2022 to August 2022. The objective of the pilot phase was to develop and validate the tool for measuring social comparison as well as to determine the feasibility of the study. Because of the lack of availability of scales for measuring Social Comparison in the Indian context, the present scale was developed by the author to fit the population of study. We have developed Instagram as a tool for Social Comparison, keeping the original scale as a framework. The original scale is called as Extent Thoughts Measure developed by Gayle R Bessenoff in 2006 for their study purpose. Permission was obtained to use the tool in our research. However, we have not used that scale nor borrowed any questions from it. This phase included constructing items for Social Comparison and Instagram Use Scale, obtaining face validity from experts of the field and selecting participants and administering all the three scales. The participants were approached through various mediators such as friends, acquaintances and family via online platforms. The participants were selected based on the inclusion and exclusion criteria through online mode using google forms Consent form was given, which was filled by the participants through online mode where they were asked to mark a tick if they agree to take part in the study by saying ‘I agree’ or mark a tick if they disagree to take part in the study by saying ‘I disagree.’ Only the participants who chose the ‘I agree’ option were considered for the study. A socio-demographic sheet was provided to the study participants. Online survey forms were administered containing the three questionnaires used. Clear and detailed instructions were given before every questionnaire. The responses were collected according to mutually set period by researcher and participants. For the pilot phase, 10 samples were recruited according to the inclusion criteria of the study. Through the use of Instagram as a Tool for Social Comparison Scale, their total scores for Social Comparison were obtained. The typical score of Social Comparison in the population being studied was found to be 23 (M=24). This shows that level of Social Comparison among the population being studied was slightly below average. In the pilot phase we had also administered other two research scales (Body Self Image Questionnaire Short Form and Exercise Motivation Inventory) to check the duration required and check the feasibility of the study. The study was found to be feasible in terms of duration, accessibility of the population and instruments required. The items of the questionnaire were found to be appropriate for the population. The population was found to be relatively accessible to the researcher, and participants were recruited mainly through email and other social media. The questionnaires required around 20 minutes to complete, and participants had no issues completing them as required. No modifications were made to the research instruments or the research design after the pilot study.

*Main phase -* The main study took place from September 2022 to January 2023. After the Institutional Ethical Committee clearance, the data collection for the study took place. The participants were selected based on the inclusion and exclusion criteria through online mode using google forms. The participants selected mainly included friends, acquaintances and family who satisfy the inclusion criteria. Consent form was given to be filled by the participants. Consent form was given, which was filled by the participants through online mode where they were asked to mark a tick if they agree to take part in the study by saying ‘I agree’ or mark a tick if they disagree to take part in the study by saying ‘I disagree.’ Only the participants who chose the ‘I agree’ option were considered for the study. A socio-demographic sheet was provided to the study participants and online survey forms were administered containing the three questionnaires used. Clear and detailed instructions were given before every questionnaire. The responses were collected according to mutually set period by researcher and participants.

### Data analysis

Version 25.0 of IBM SPSS Statistics for Windows. IBM Corp., Armonk, New York, was employed to examine the acquired data ( IBM Corp., 2017). We used the necessary tables to express our results as proportions. In our study, the normality of the variables was assessed. Shapiro-Wilk statistic, Q-Q plots, histograms were used to evaluate the normality of the variables. In this study, Sex and Instagram use are the independent variables; body image, motivation to exercise, and social comparison are the dependent variables. The data for the independent variable sex showed a normal distribution with a mean (M) of 3.43, a standard deviation (SD) of 1.50, and a Shapiro-Wilk score of 0.845 (>0.05). The Shapiro-Wilk test result (p = 0.091) for the dependent variable body image indicates that the data is normally distributed. This is corroborated by descriptive statistics (mean = 73.25, median = 73.00), and also visual inspections using Q-Q plots and histograms. In terms of another study variable social comparison, the Q-Q plot displays data points that are nearly aligned with the diagonal, and the descriptive statistics (mean = 24.96, median = 25.00), skewness = -0.063, kurtosis = -0.382, and Shapiro-Wilk test result (p = 0.184) reveal a normal distribution. The Shapiro-Wilk test (W = 0.968, p < 0.001) suggested a significant deviation from normalcy, but the Kolmogorov-Smirnov test (D = 0.0853, p = 0.093) did not show a significant deviation; visual inspections revealed minor deviations, but the assumption of normalcy is deemed met because of the bigger sample size (N = 211) and the near-normal appearance in visual inspections. Overall, it was determined that parametric tests may be appropriately applied to the data in this study due to these findings and the sizable sample size.

Based on this normality test, parametric tools were used for the analysis. In order to find the difference between two groups with respect to the sociodemographic and dependent variables, independent sample t-test was used to find the Sex differences in body image, exercise motivation and social comparison. Univariate analysis of variance (ANOVA) was used to test the difference between two or more groups with respect to the sociodemographic and the dependent variables such as between body image and people who exercised regularly, not regularly and sometimes.

## Results

### Descriptive statistics

*Characteristics of the sample*

A total of 253 participants were recruited for the study. The participants who weren’t active users of Instagram and who had a past history of physical/psychiatric illness were excluded. Thus, the final sample consisted of 212 participants (males = 106, females = 106) aged between 20 and 30 years with mean age of males, M = 22.76, and standard deviation SD = 1.84 and mean age of females, M = 23.05 and standard deviation SD = 2.21. The data was normally distributed with Shapiro-Wilk statistic being 0.845 (>0.05) and mean M = 3.43 and standard deviation SD = 1.50.

### Instagram use

All the participants of the study were active Instagram users. Table 1 describes the Mean and standard deviation of Instagram usage characteristics of the sample. Their median use of Instagram was one hour per day. They reported following a median number of more than five celebrities and a median number of one-five fitness related pages. The participants also reported how frequently they watched fitness related images and videos and how frequently they posted fitness related images and videos. 15.4% of the participants reported watching fitness related posts daily, 29.2% (weekly), 17% (monthly) and 38.3% never watched fitness related posts. Similarly, 82.6% of the participants reported that they never posted pictures/videos related to fitness, 10.7% (monthly), 3.2% (weekly) and 3.6% (daily).

**Table 1.** Mean and standard deviation of Instagram usage characteristics of the sample.

Socio-demographics	Mean	Standard Deviation
Time spent on Instagram (Hours)	2.03	0.81
Number of celebrities followed on Instagram	8.4	2.33
Duration of exercise (Hours)	1.21	0.64
Number of fitness related pages followed on Instagram	2.18	1.74

N = 212 (Males = 106, Females = 106).

As can be seen in Table 2, mean scores for both Body Image (M = 73.61.84, SD = 13.66) and Social Comparison (M = 25.60, SD = 6.24) were found to be higher among females. In contrast, mean scores for Exercise Motivation was found to be higher among males (M = 156.36, SD = 43.32).

**Table 2.** Mean and standard deviation of the variables Body Image, Exercise Motivation and Social Comparison in males and females in the age group 20-30years.

Variable		Mean	SD
Body Image	Male	68.84	15.58
	Female	73.61	13.66
Exercise Motivation	Male	156.36	43.32
	Female	136.38	56.93
Social Comparison	Male	24.33	7.39
	Female	25.60	6.24

### Inferential statistics

*Sex differences in body image*

Independent samples *t test* was conducted to compare the Sex differences in body image. As displayed in Table 3, there was significant difference in body image (t (2) = 0.019, p = 0.05) with mean score of females (M = 73.61, SD = 13.66), which was found to be higher than males (M = 68.84, SD = 15.58). The magnitude of the differences in the means (mean difference = -4.77, 95% *CI:* -8.77 to -0.77). Thus, there is a significant difference in body image between males and females, with mean body image higher among females in the age group 20-30 in the present study.

**Table 3.** Sex difference in Body Image, Exercise Motivation and Social Comparison among the study sample.

Variable	Group	Levene’s test for equality of variances							t-test for equality of means		
		Mean	SD	F	p value	t-test	df	Sig. (2 tailed)	Mean Difference	Standard Error Difference	95% Confidence Interval of the Difference
											Lower Upper
Body Image	Male	68.84	15.58								
				1.948	0.164	-	2	0.019	-4.77	2.02	-8.77 -0.77
	Female	73.61	13.66			2.355					
Exercise Motivation	Male	156.36	43.32								
				7.948	0.005	-2.849	2	0.005	-19.97	7.01	-33.79 -6.15
	Female	136.38	56.93								
Social Comparison	Male	24.33	7.39								
				3.251	0.073	-1.345	2	0.180	-1.27	0.94	-3.14 0.59
	Female	25.60	6.24								

SD = Standard Deviation, F = F Statistic, P = Probability value, df = degrees of freedom, Sig. = Significant.

*Sex differences in exercise motivation*

Independent samples *t test* was conducted to compare the Sex differences in exercise motivation. As displayed in Table 3, there was significant difference in exercise motivation (t(2) = 0.005, p = 0.05) with mean score for males (M = 156.36, SD = 43.32), which was found to be higher than females (M = 136.38, SD = 56.93). The magnitude of the differences in the means (mean difference = -19.97, 95% *CI:* -33.79 to -6.15). Thus, there is a significant difference in exercise motivation between males and females, with mean exercise motivation higher among males in the age group 20-30 in the present study.

*Sex differences in social comparison*

Independent samples *t test* were conducted to compare the Sex differences in social comparison. As displayed in Table 3, no significant difference in social comparison across gender was seen in the present study.

*Difference in body image between people who followed fitness related pages on Instagram and those who did not.*

Independent samples *t test* was conducted to compare the difference in body image across people who followed fitness related pages with those who did not. As displayed in Table 4, there was significant difference in body image (t(2) = 0.028, p = 0.05) with mean score for people who follow fitness related pages (M = 72.63, SD = 13.44), which was found to be higher than people who did not follow fitness related pages on Instagram (M = 69.44, SD = 16.21). The magnitude of the differences in the means (mean difference = 3.19, 95% *CI:* -0.84 to 7.22). Thus, there was a significant difference in body image between people who followed fitness related pages on Instagram and those who did not, with mean body image higher among people who followed fitness related pages on Instagram.

**Table 4.** The difference in body image between people who followed fitness related pages on Instagram and those who did not.

Component	Group					Levene’s Test for Equality of Variances				t-test for Equality of Means	
Whether fitness-related pages followed on Instagram		Mean	SD	F	Sig.	t	df	Sig. (2 tailed)	Mean Difference	Std. Error Difference	95% Confidence Interval of the Difference
											Lower Upper
	Yes	72.63	13.44								
				4.899	0.028	1.560	2	0.120	3.19	2.04	-0.84 7.22
	No	69.44	16.21								

SD = Standard Deviation, F = F Statistic, t = test statistic, df = degrees of freedom, Sig. = Significant.

Difference in body image between people who exercised regularly and those who did not.

Table 5 represents the difference between people who exercise regularly and body image in males and females in the age group 20-30. One way ANOVA test enumerated in the table revealed a significant difference in body image between people who exercise regularly and those who did not at p = 0.05 in the present study.

**Table 5.** Difference in body image between people who exercised regularly and those who did not.

Variable	N	Mean	SD	df	F	Sig.
Do you exercise regularly?						
Yes	63	76.37	13.73	2	11.268	0.018
No	60	64.36	14.63			
Sometimes	89	72.16	14.12			

SD = Standard Deviation, N = Sample size, F = F Statistic, df = degrees of freedom, Sig. = Significant.

Table 6 shows the frequency and percentage of the extrinsic and intrinsic motivational factors for exercise in men. Among the dimensions, majority of men reported ill-health avoidance & positive health (98.11%), revitalization (96.22%), strength & endurance (89.62%), stress management (86.79%), challenge & appearance (81.13%) as motivators for their exercise behaviours.

**Table 6.** Frequency and percentage of the extrinsic and intrinsic motivational factors for exercise in men.

Motivational factors (subscale)	Number of items	Sample items	Frequency	Percentages
Stress management	4	Because it helps reduce tension	92	86.79
Revitalization	3	Because it makes me feel good	102	96.22
Enjoyment	4	Because I enjoy the feeling of exerting myself	83	78.30
Challenge	4	To give me goals to work toward	86	81.13
Social recognition	4	To show my worth to others	62	58.49
Affiliation	4	To spend time with friends	64	60.37
Competition	4	Because I like trying to win in PA	59	55.66
Health pressures	3	Because my doctor advised me to exercise	62	58.49
Ill-health avoidance	3	To prevent health problems	104	98.11
Positive health	3	To have a healthy body	104	98.11
Weight management	4	To stay slim	77	72.64
Appearance	4	To look more attractive	86	81.13
Strength & endurance	4	To increase my endurance	95	89.62
Nimbleness	3	To stay/become more agile	84	79.24

## Discussion

The goal of the present study was to examine the Sex difference in body image, exercise motivation and social comparison among people who use Instagram in the age group 20-30. The current study advances our understanding of how Instagram use affects both men and women in the age range 20-30 when it comes to body image, exercise motivation, and social comparison. It is significant because it broadens the study of mass media to include Instagram, a medium for social media with a strong visual component. Due to the majority of studies’ attention being directed towards women, there is relatively little information available on the influence of social networking sites on men’s body image. The perfect male figure, however, has also received attention over the past few decades. As evidenced by images in magazines or action figures, the idealized concept of a more slender, muscular, and V-shaped male body has begun to gain attraction among Westerners ( Voges *et al*., 2019). As a result, men, these days are also forced to work towards building an ideal body that is challenging to achieve. We chose to focus on Instagram in particular because a large body of studies on social media and body image has been focused on social networking sites like Facebook, Twitter etc. Since Instagram is one of the most widely utilized social media platforms (SNS) in use today, it is crucial to pay more attention to its influence. The first hypothesis was that there were no Sex differences in body image, Secondly, it was hypothesized that there were no Sex differences in exercise motivation. Finally, it was hypothesized that there were no Sex differences in social comparison in males and females Instagram users in the age group 20-30.

The first major finding of the study was that levels of body image concerns were higher among females, which was supported by a vast array of previous literature. Previous research findings suggested that ideal form of women has evolved through time, as women are frequently shown to be underweight in magazines and advertisements ( Luff & Gray, 2009). Multiple research projects have indicated that there is no doubt that a constant encounter with idealized body types has harmful effects, low self-esteem, and body dissatisfaction, even though studies are hesitant to make that assertion ( Bessenoff, 2006). Women are constantly met with the need to “control” their body weight below the normal, expected, healthy range as an attempt to fit into the society’s portrayal of idealized women’s body. This has been demonstrated by previous research that explored the relationship between social media and women’s body image, which revealed that, pre-exposure to social media photographs of fashion models increased women’s’ drive for thinness, which was correlated with post-exposure low mood and dissatisfaction with their body image ( Drames, 2016). These findings imply that regardless of the setting or individual depicted, exposure to “thin” ideal images that are frequently encountered, either through newspapers, magazines, social media, or peers may have a comparable detrimental impact on women’s body satisfaction. Future studies might specifically investigate this proposition.

The finding that body image concerns were higher among women strongly supported another major finding of the study, which showed that a significant difference exists in body image across gender; with higher body image concerns among females. This finding was supported by numerous previous research which indicated that women oftentimes feel a gap between their existing real bodies and the elusive - ideal female body, which eventually paves the way for body dissatisfaction ( Mills *et al*., 2018). Compared to boys, girls are more conscious about the ways in which their body weight influences their appearance, beginning right from childhood ( Shriver *et al.,* 2013b). In addition, women are inclined to have decreased self-esteem when they feel that their weight is above the idealized weight norms, whereas men tend to experience this only when they feel that they are obese ( Shriver *et al*., 2013a). Congruent to this finding, it was identified that girls give importance to beauty and aesthetics and place less emphasis on the functioning capacity of their bodies than did boys ( Abbott & Barber, 2010). More so than the idealized male masculinity, which is represented by more varied media depictions, the norms for the female body ideal appear to be more well defined ( Boute *et al*., 2011).

The second major finding was that the levels of exercise motivation was found to be higher among males. This was seen in the data from the present study which indicated that men reported on an average tending to spend more time on exercise and other physical activities than women. The socio-demographic data also revealed that as compared to women, men are more inclined to spend their time in the gym (30%). The dimensions of the Exercise Motivation Inventory used in this study indicated that men on an average were motivated to exercise more as compared to women due to many reasons, including the need to gain Strength & Endurance, Ill-health Avoidance, Revitalization, Challenge, Enjoyment, Stress Management. These findings have been supported by previous research findings suggesting gender disparities in exercise involvement ( Bauman *et al*., 2009). In traditional eastern cultures, where women typically take on the role of carers for the family and have less time for exercise pointing that, gender roles may have an impact on regular exercise habit ( Craft, Carroll & Lustyk, 2014). Thus, even though women have body image issues due to social media exposure, they might be less indulgent in exercise behaviours due to these reasons ( Chen *et al*., 2011). Additionally, these findings have also been strengthened by previous literature exploring the effect of social media on exercise behaviours in men ( Rote *et al*., 2015). The success of these platforms has been linked to a number of factors, consisting of the sense of belonging fostered by physical activity-based group chats, which increased accountability for daily exercise, social media-provided encouragement and praise, motivation gained from knowing other members’ progress on exercise behaviours, and motivation gained from viewing posts regarding the advantages of exercising ( Chen *et al*., 2011).

This result provides strong evidence for another major finding from the present study which revealed that a significant difference in exercise motivation across gender with higher exercise motivation in males in the present study. Previous research which focused on studying gender disparities in the motivations for exercising supports this finding ( Markland & Tobin, 2010). It is likely that men and women may feel distinct advantages from exercise as a result of their activity attributions given these gender disparities in reasons for exercising. Men in general are more likely to attribute exercise to social and competitive factors, while women are more likely to attribute exercise to appearance-related factors, such as to lose or maintain weight. Furthermore, it was observed that rather than exercise itself, women’s reasons for exercising predicted their quality of life. Engaging in physical activity with the goal of getting fit or reducing weight, for instance, was linked to poor quality of life, whereas engaging in physical activity to uplift mood or enhance health was linked to an increased quality of life ( Craft *et al*., 2014). Previous research also indicates that, men and women “enjoy” exercise differently. This was visible in the mental health benefits derived from physical activity. Men who engaged in vigorous physical activity reported lower levels of depressive and anxiety symptoms as well as other visible signs of mental stress ( Rote *et al*., 2015). Men may experience less harm to their mental health as a result of their higher levels and more intense physical activity ( Craft, Carroll & Lustyk, 2014) It was determined that men benefit more from intensive exercise than women do from lighter exercise ( Asztalos *et al*., 2010). Additionally, these findings have also been strengthened by previous literature exploring the effect of social media on exercise behaviours in men. The success of these platforms (Facebook, Instagram, Snapchat and other online platforms) has been attributed to a number of factors, including the sense of belonging fostered by physical activity-based group chats, which increased accountability for daily exercise, social media-provided encouragement and praise, motivation gained from knowing other members’ progress on exercise behaviours, and motivation gained from viewing posts regarding the advantages of exercising ( Jones, 2001; Bessenoff, 2006).

With respect to social comparison, the results revealed that the levels of comparison were slightly higher among women as compared to men. This is congruent with a past quantitative data analysis research, which suggested that internalization of a “thin” ideal is facilitated by a strong inclination for evaluative comparisons with appealing targets ( Morrison, Kalin & Morrison, 2004; Schutz, Paxton & Wertheim, 2002) and mediates the impact of the media on body dissatisfaction in women ( Van den Berg *et al*., 2002). Studies have shown that women usually make comparison of their body with attractiveness, whereas men tend to compare their body in terms of build. Because women are frequent targets of ideal body advertisements and commercials in general media as well as social media, these results are not surprising. But this doesn’t deny the fact that men too are confronted with unrealistic comparisons aided by media. In fact, a qualitative study indicated that male participants reported that they felt forced by the social networking sites to appear more masculine, particularly on the upper body ( Ridgeway & Tylka, 2005). Thus, the levels of social comparison being more or less similar in males and females indicated that both sexes are confronted with self-evaluation with regards to media portrayals of ideal men and women, which might also be a predictor of body image issues in this population. This calls for a more in-depth investigation in future to better understand the mediating effect of social comparison on body image in both men and women.

The finding that there were no Sex differences in social comparison supported the above finding and was incongruent with previous research. Literature has consistently shown that even though men and women frequently coexist in the same social environments, there is ample proof that even the identical social situations can be interpreted and experienced differently by men and women ( Grabe, Ward *et al*. 2008). Women were shown to be increasingly prone to view themselves from an external point of view and participate in a self-critical comparison process regarding their bodies because of the cultural preoccupation on ideal female body as an aesthetic object relative to the male body ( Franzoi *et al*., 2012). In contrast, men were shown to be more optimistic and less judgmental of themselves when assessing their bodies than women ( Miller & Ross, 1975). However, present study could not find any difference in social comparison across gender. This points to the assumption that there could be other factors such as the sample size, sociodemographic characteristics that might have played a role in producing these results.

### Theoretical implications

According to Self-Determination Theory (SDT), motivation is divided into intrinsic and extrinsic. According to SDT, intrinsic motivation generally leads to more sustained involvement in behaviours such as exercise. There is strong evidence, supporting SDT's usefulness to understanding exercise motivation according to Teixeira *et al.* (2012). They conducted a systematic study that found strong evidence supporting SDT's usefulness to understanding exercise motivation. Key findings show that autonomous types of motivation, whether driven by intrinsic motivation (enjoyment, personal challenge) or recognized regulation (valuing health benefits), are consistently associated with higher levels of physical activity. This review particularly focuses on sex differences in exercise motivation. According to the review, increased exercise motivation in men is primarily driven by intrinsic attributes such as strength and endurance, emphasizing the necessity of improving intrinsic motivation in exercise promotion tactics. On the other hand, for women, body dissatisfaction appears to be a strong motivation for exercise. In women, exercise motivation is frequently linked to extrinsic reasons such as attractiveness objectives. This emphasizes the need for gender-specific approaches to encouraging exercise. The current study implication also emphasizes on considering sex difference for encouraging the exercise. For example, addressing body image issues and fostering a supportive environment that shifts the focus away from beauty and toward overall well-being may be beneficial for women. On the other hand, strategies that increase intrinsic motivations, such enjoyment of oneself and skill development, may be more beneficial for men.

### Implications

The present study contributes to the literature on social media and body image, exercise motivation and social comparison, considering the importance of increased use of SNS such as Instagram over the recent years. This study revealed that body image concerns were higher among women. This finding suggests that women should be educated about the harmful effects of body image distortions and resulting lowered self-esteem and body dissatisfaction. The finding that drive for exercise was higher among males provides a foundation for understanding the motivators for male exercise behaviours including the genetic, psychological, social and cultural factors. We need to enhance the intrinsic motivational factors in men who exercise regularly to the extent that they get to experience a sense of autonomy, competence and relatedness (in line with the self-determination theory) by seeing the difference for themselves, rather than driven by external standards of body building and fitness portrayed in social media. With respect to women’s body image issues, we need to delve into the sociocultural factors including parenting styles, academic settings, and other possible contexts where they could have internalized these unrealistic standards about their own body. It all comes to a single inference that the more you keep comparing yourself, the more these cognitive distortions gets deeply ingrained in your schema. So, the key is to break the chain very early in the critical developmental stages which can be accomplished through parental psychoeducation, showing the harsh reality of the media and strengthening healthy relationships. Body image interventions: Targeted interventions for women are essential to mitigate the negative impact of social media on body satisfaction. Programs should focus on enhancing media literacy, promoting body positivity, and encouraging critical thinking about media messages. This information can aid in planning interventions for mitigating the potential negative effects of exercise behaviours such as exercise addiction, however more in-depth research is needed.

*Strengths, limitations and future research*

It was a strength of the current study that it collected a sample that gave disparate information about the participants’ Instagram activity including the duration of use, celebrities followed, fitness related pages followed, and frequency of sharing posts/videos related to fitness which gave a clearer picture of how these factors could be a contributing factor to the Sex differences in the variables studied.

Despite these strengths, there were a few limitations that ought to be mentioned and which may help to direct future study. First, the study’s use of a purposive sampling method poses a significant restriction. The representativeness of Indian Instagram users may still be overestimated even if the sample comprises of 212 respondents chosen from various online platforms. The study findings can be generalized to populations in the similar age range and similar sociocultural milieu. However, it is recommended that future studies can incorporate a larger sample size. Another limitation was that although a significant difference in body image and exercise motivation was observed across gender, the huge community of SNS users with over 1.3 billion users on Instagram indicates that these discoveries are relevant only at a population level. The study’s design constituted still another drawback. Its cross-sectional design prevents us from understanding the causal connection between the variables. Finally, it is impossible to completely rule out the possibility of residual confounding such as simultaneous use of other SNS platforms and social desirability bias in self-reported data. There was no supplemental information collected from other potential sources, such as relatives or peers.

Given these limitations, future research can focus on exploring individuals with a larger range of ages, given that older and middle-aged people engage in social media, although in a smaller amount than younger people. All future studies should be proactive to recruit culturally diverse samples as often as possible to be able to draw conclusions about the potentially differential effects as well as to make comparisons across individuals from diverse backgrounds. Future research can also delve deeper into the sociodemographic factors explored in the study to better understand how it can play a role in Sex difference in these variables with large samples. Also, experimental and longitudinal studies are recommended in future studies, that may examine the cause-effect relationships among variables. In future studies, additional data from other possible sources such as family members, peers, or significant others in the environment may be collected to measure.

## Conclusions

Body image, exercise motivation, and social comparison are interrelated concepts that shape the way individuals perceive themselves and others. According to this study, women were more likely than men to have body image concerns. This finding suggests that women need to be made aware of the risks associated with distorted body images, low self-esteem, and body dissatisfaction. The discovery that males were more motivated to exercise than females lays the groundwork for understanding the genetic, psychological, social, and cultural motivations for male exercise behaviours. Planning interventions to lessen the possible negative effects of exercise behaviours, such as exercise addiction, can be made easier with the help of this information. It is critical to identify who is at risk of acquiring dangerous exercise practises as a result of a change in their body image for both prevention and treatment. The study’s findings will aid in this knowledge.

## Data Availability

Open Scientific Framework: Sex difference in body image, exercise motivation and social comparison among Instagram users: a cross sectional study. DOI: https://doi.org/10.17605/OSF.IO/S6R4T ( Vasudha & Nimiya, 2023) This project contains the following underlying data:
• Instagram as a tool for social comparison responses.xlsx • BSIQ responses.xlsx (Responses of all the participants on Body Self Image Questionnaire) • EMI 2 responses.xlsx (Exercise Motivation Inventory participant’s response) This dataset contains the following extended data:
• INSTAGRAM AS A TOOL FOR SOCIAL COMPARISON.docx • Scale validation.pdf (Instagram as a tool for social comparison scale validation) • Study flow figure.jpg •
STROBE_checklist_cross-sectional-F1000.docx Data is available under the terms of the Creative Commons Attribution 4.0 International license (CC-BY 4.0).

## References

[ref1] Abbott BD Barber BL : Embodied image: Sex differences in functional and aesthetic body image among Australian adolescents. *Body Image.* 2010;7(1):22–31. 10.1016/j.bodyim.2009.10.004 19945925

[ref2] Al-Menayes JJ : Dimensions of social media addiction among university students in Kuwait. *Psychol. Behav. Sci.* 2015;4(1):23–28. 10.11648/j.pbs.20150401.14

[ref3] Asztalos M De Bourdeaudhuij I Cardon G : The relationship between physical activity and mental health varies across activity intensity levels and dimensions of mental health among women and men. *Public Health Nutr.* 2010;13(8):1207–1214. 10.1017/S1368980009992825 20018121

[ref4] Bauman A Bull F Chey T : The international prevalence study on physical activity: results from 20 countries. *Int. J. Behav. Nutr. Phys. Act.* 2009;6(1):1–11. 10.1186/1479-5868-6-21 19335883 PMC2674408

[ref5] Bessenoff GR : Can the media affect us? Social comparison, self-discrepancy, and the thin ideal. *Psychol. Women Q.* 2006;30(3):239–251. 10.1111/j.1471-6402.2006.00292.x

[ref6] Brown Z Tiggemann M : Attractive celebrity and peer images on Instagram: Effect on women’s mood and body image. *Body Image.* 2016;19:37–43. 10.1016/j.bodyim.2016.08.007 27598763

[ref7] Buote VM Wilson AE Strahan EJ : Setting the bar: Divergent sociocultural norms for women’s and men’s ideal appearance in real-world contexts. *Body Image.* 2011;8(4):322–334. 10.1016/j.bodyim.2011.06.002 21775228

[ref8] Chatzopoulou E Filieri R Dogruyol SA : Instagram and body image: Motivation to conform to the “Instabod” and consequences on young male wellbeing. *J. Consum. Aff.* 2020;54(4):1270–1297. 10.1111/joca.12329

[ref9] Chen YJ Huang YH Lu FH : The correlates of leisure time physical activity among an adults population from southern Taiwan. *BMC Public Health.* 2011;11:1–9. 10.1186/1471-2458-11-427 21639878 PMC3141445

[ref10] Cohen R Newton-John T Slater A : The relationship between Facebook and Instagram appearance-focused activities and body image concerns in young women. *Body Image.* 2017;23:183–187. 10.1016/j.bodyim.2017.10.002 29055773

[ref11] Craft BB Carroll HA Lustyk MKB : Sex differences in exercise habits and quality of life reports: assessing the moderating effects of reasons for exercise. *Int. J. Lib. Arts Soc. Sci.* 2014;2(5):65–76. Reference Source 27668243 PMC5033515

[ref13] De Vries DA Möller AM Wieringa MS : Social comparison as the thief of joy: Emotional consequences of viewing strangers’ Instagram posts. *Media Psychol.* 2018;21(2):222–245. 10.1080/15213269.2016.1267647

[ref14] Eckler P Kalyango Y Paasch E : Facebook use and negative body image among US college women. *Women Health.* 2017;57(2):249–267. 10.1080/03630242.2016.1159268 26933906

[ref15] Fardouly J Diedrichs PC Vartanian LR : Social comparisons on social media: The impact of Facebook on young women’s body image concerns and mood. *Body Image.* 2015;13:38–45. 10.1016/j.bodyim.2014.12.002 25615425

[ref16] Franzoi SL Vasquez K Sparapani E : Exploring body comparison tendencies: Women are self-critical whereas men are self-hopeful. *Psychol. Women Q.* 2012;36(1):99–109. 10.1007/s11199-006-9162-4

[ref17] Grabe S Ward LM Hyde JS : The role of the media in body image concerns among women: a meta-analysis of experimental and correlational studies. *Psychol. Bull.* 2008;134(3):460–476. 10.1037/0033-2909.134.3.460 18444705

[ref18] Gültzow T Guidry JP Schneider F : Male body image portrayals on instagram. *Cyberpsychol. Behav. Soc. Netw.* 2020;23(5):281–289. 10.1089/cyber.2019.0368 32286866

[ref19] Halder S Mondal P : Body Image and the Preference of Exercise in Everyday Life: A Studyon Young Generation. *IOSR Journal of Humanities and Social Science (IOSR-JHSS).* 2020;25(11):12–16. Reference Source

[ref20] Hobza CL Walker KE Yakushko O : What about men? Social comparison and the effects of media images on body and self-esteem. *Psychol. Men Masculinity.* 2007;8(3):161–172. 10.1037/1524-9220.8.3.161

[ref21] Holland G Tiggemann M : A systematic review of the impact of the use of social networking sites on body image and disordered eating outcomes. *Body Image.* 2016;17:100–110. 10.1016/j.bodyim.2016.02.008 26995158

[ref22] IBM Corp: *IBM SPSS Statistics for Windows, Version 25.0.* Armonk, NY: IBM Corp;Released 2017.

[ref23] Jones DC : Social comparison and body image: Attractiveness comparisons to models and peers among adolescent girls and boys. *Sex Roles.* 2001;45:645–664. 10.1177/2056305116640559

[ref24] Kemp S. Digital 2023: Global Overview Report:2023. Reference Source

[ref25] Lewallen J Behm-Morawitz E : Pinterest or thinterest?: Social comparison and body image on social media. *Social media+ society.* 2016;2(1):205630511664055. 10.1177/2056305116640559

[ref26] Littrell A : The Relationship Between Body Image and Exercise Type. 2017. Reference Source

[ref27] Luff GM Gray JJ : Complex messages regarding a thin ideal appearing in teenage girls’ magazines from 1956 to 2005. *Body Image.* 2009;6(2):133–136. 10.1016/j.bodyim.2009.01.004 19250889

[ref28] Markland D Hardy L : The Exercise Motivations Inventory: Preliminary development and validity of a measure of individuals’ reasons for participation in regular physical exercise. *Personal. Individ. Differ.* 1993;15(3):289–296. 10.1016/0191-8869(93)90219-S

[ref29] Markland D Ingledew DK : The measurement of exercise motives: Factorial validity and invariance across gender of a revised Exercise Motivations Inventory. *Br. J. Health Psychol.* 1997;2:361–376. 10.1111/j.2044-8287.1997.tb00549.x

[ref30] Markland D Tobin VJ : Need support and behavioural regulations for exercise among exercise referral scheme clients: The mediating role of psychological need satisfaction. *Psychol. Sport Exerc.* 2010;11(2):91–99. 10.1016/j.psychsport.2009.07.001

[ref31] Miller DT Ross M : Self-serving biases in the attribution of causality: Fact or fiction? *Psychol. Bull.* 1975;82(2):213–225. 10.1037/h0076486

[ref32] Mills JS Musto S Williams L : “Selfie” harm: Effects on mood and body image in young women. *Body Image.* 2018;27:86–92. 10.1016/j.bodyim.2018.08.007 30149282

[ref33] Morrison TG Kalin R Morrison MA : BODY-IMAGE EVALUATION AND BODY-IMAGE AMONG ADOLESCENTS: A TEST OF SOCIOCULTURAL AND SOCIAL COMPARISON THEORIES. *Adolescence.* 2004;39(155):571–592. 15673231

[ref34] Panjrath MY Tiwari S : “Why Them, Not Me?”: A Study Exploring the Impact of Following Fashion Influencers on Instagram on Body Image Satisfaction of Adolescent Girls and Middle-aged Women. *Int. J. Psychosoc. Rehabil.* 2021;25(02). 10.37200/IJPR/V2512/PR320040

[ref51] Prichard I Kavanagh E Mulgrew KE : The effect of Instagram# fitspiration images on young women’s mood, body image, and exercise behaviour. *Body Image.* 2020;33:1–6. 10.1016/j.bodyim.2020.02.002 32062021

[ref35] Ridgeway RT Tylka TL : College Men’s Perceptions of Ideal Body Composition and Shape. *Psychol. Men Masculinity.* 2005;6(3):209–220. 10.1037/1524-9220.6.3.209

[ref36] Rote AE Klos LA Brondino MJ : The efficacy of a walking intervention using social media to increase physical activity: a randomized trial. *J. Phys. Act. Health.* 2015;12(s1):S18–S25. 10.1123/jpah.2014-0279 25599378

[ref37] Rowe DA : Factorial validity and cross-validation of the Body Self-Image Questionnaire (Short Form) in young adults. *Health.* 2005;73(81):49.

[ref38] Schutz HK Paxton SJ Wertheim EH : Investigation of body comparison among adolescent girls 1. *J. Appl. Soc. Psychol.* 2002;32(9):1906–1937. 10.1111/j.1559-1816.2002.tb00264.x

[ref39] Scirrotto Drames T : The Impact of Internet Social Networking on Young Women’s Mood and Body Image Satisfaction: An Experimental Design. 2016. Reference Source

[ref40] Shriver LH Betts NM Wollenberg G : Dietary intakes and eating habits of college athletes: are female college athletes following the current sports nutrition standards? *J. Am. Coll. Heal.* 2013a;61(1):10–16. 10.1080/07448481.2012.747526 23305540

[ref41] Shriver LH Harrist AW Page M : Differences in body esteem by weight status, gender, and physical activity among young elementary school-aged children. *Body Image.* 2013b;10:78–84. 10.1016/j.bodyim.2012.10.005 23228485

[ref42] Singh MM Parsekar SS Bhumika TV : Body image, eating disorders and role of media among Indian adolescents. *J. Indian Assoc. Child Adolesc. Ment. Health.* 2016;12(1):9–35. 10.1177/0973134220160102

[ref43] Statista Research Department: Distribution of Instagram users worldwide as of January2022, by age group. United States. 2022. Reference Source

[ref44] Talbot D Saleme D : Evidence of attentional bias toward body stimuli in men. *Atten. Percept. Psychophys.* 2022;84(4):1069–1076. 10.3758/s13414-022-02466-7 35355232 PMC9076707

[ref52] Teixeira PJ Carraça EV Markland D : Exercise, physical activity, and self-determination theory: A systematic review. *Int. J. Behav. Nutr. Phys. Act.* 2012;9(1):78. 10.1186/1479-5868-9-78 22726453 PMC3441783

[ref45] Van den Berg P Thompson JK Obremski-Brandon K : The tripartite influence model of body image and eating disturbance: A covariance structure modeling investigation testing the mediational role of appearance comparison. *J. Psychosom. Res.* 2002;53(5):1007–1020. 10.1016/S0022-3999(02)00499-3 12445590

[ref46] Vogel EA Rose JP Okdie BM : Who compares and despairs? The effect of social comparison orientation on social media use and its outcomes. *Personal. Individ. Differ.* 2015;86:249–256. 10.1016/j.paid.2015.06.026

[ref47] Voges MM Giabbiconi CM Schöne B : Sex differences in body evaluation: Do men show more self-serving double standards than women? *Front. Psychol.* 2019;10:544. 10.3389/fpsyg.2019.00544 30930819 PMC6428027

[ref48] Von Elm E Altman DG Egger M : The Strengthening the Reporting of Observational Studies in Epidemiology (STROBE) statement: guidelines for reporting observational studies. *Lancet.* 2007;370(9596):1453–1457. 10.1016/S0140-6736(07)61602-X 18064739

[ref49] Yang CC : Instagram use, loneliness, and social comparison orientation: Interact and browse on social media, but don’t compare. *Cyberpsychol. Behav. Soc. Netw.* 2016;19(12):703–708. 10.1089/cyber.2016.0201 27855266

[ref50] Vasudha KG Nimiya A : Sex difference in body image, exercise motivation and social comparison among Instagram users: a cross sectional study (new). 2023, July 17. 10.17605/OSF.IO/S6R4T PMC11220442 38962298

