# Supplementary material for: Sex difference in body image, exercise motivation and social comparison among Instagram users: a cross sectional study
Source: F1000Res. 2024 Jun 14;12:1506. Originally published 2023 Nov 27. [Version 2] doi: 10.12688/f1000research.134799.2 (PMC11220442; doi:10.12688/f1000research.134799.2)
Supplement: Peer review history

# Reviewer report: Reviewer response for version 2

**DOI:** 10.5256/f1000research.167811.r291226

**Published:** 2024-07-02

**Authors:** Niyaz Panakaje, S. M. Riha Parvin

**Affiliations:** 1 Yenepoya University, Mangalore, Karnataka, India; 2 IMC, Srinivas University, Mangaluru, Karnataka, India

No further comments to make

 Niyaz Panakaje

 YEN-REFINED, Yenepoya (Deemed to be University), Mangalore

Is the work clearly and accurately presented and does it cite the current literature?

Yes

If applicable, is the statistical analysis and its interpretation appropriate?

Yes

Are all the source data underlying the results available to ensure full reproducibility?

Yes

Is the study design appropriate and is the work technically sound?

Partly

Are the conclusions drawn adequately supported by the results?

Yes

Are sufficient details of methods and analysis provided to allow replication by others?

Partly

Reviewer Expertise:

Higher Education, Stress, Social Media, Entrepreneurhip

We confirm that we have read this submission and believe that we have an appropriate level of expertise to confirm that it is of an acceptable scientific standard.

# Reviewer report: Reviewer response for version 2

**DOI:** 10.5256/f1000research.167811.r291227

**Published:** 2024-06-14

**Authors:** Vandana Gaur

**Affiliations:** 1 Department of Psychology, SDM Government PG College in Doiwala, Dehradun, Uttarakhand, India

The abstract effectively summarizes the study's purpose, methods, results, and conclusions. It provides a clear snapshot of the study, highlighting the key findings regarding sex differences in body image, exercise motivation, and social comparison among Instagram users.

 The methods section is well-detailed, allowing for replication of the study. The cross-sectional design is appropriate for the research questions posed. The sample size is adequate, and the recruitment methods are clearly described.

 The results are presented clearly, with appropriate use of tables and figures to illustrate key findings. The statistical analyses are robust, and the results are interpreted correctly. The study effectively highlights significant sex differences in body image, exercise motivation, and social comparison among Instagram users.

 The discussion section provides a thorough interpretation of the results, linking them back to the research questions and hypotheses. The authors discuss the implications of their findings in the context of existing literature, highlighting how their study contributes to the field.

 Overall Assessment:

 This study provides valuable insights into the sex differences in body image, exercise motivation, and social comparison among Instagram users. The methodology is sound, and the findings are clearly presented and interpreted. The paper makes a significant contribution to the literature on social media's impact on psychological and behavioral outcomes.

Is the work clearly and accurately presented and does it cite the current literature?

Yes

If applicable, is the statistical analysis and its interpretation appropriate?

Yes

Are all the source data underlying the results available to ensure full reproducibility?

Yes

Is the study design appropriate and is the work technically sound?

Yes

Are the conclusions drawn adequately supported by the results?

Yes

Are sufficient details of methods and analysis provided to allow replication by others?

Partly

Reviewer Expertise:

Social Psychology, Cognitive Psychology, Psychopathology

I confirm that I have read this submission and believe that I have an appropriate level of expertise to confirm that it is of an acceptable scientific standard.

# Response

**Published:** 2024-06-21

**Authors:** Vasudha K.G

**Affiliations:** Psychiatry, KMC Hospital Attavar, Mangaluru, Karnataka, India

Dear Ma’am,

 Thank you very much for your thoughtful and comprehensive review of our manuscript. We are pleased to hear that you found our study on “Sex differences in body image, exercise motivation, and social comparison among Instagram users” to be valuable and well-conducted.

 We appreciate your positive feedback on various aspects of our study, including the abstract, methods, results, and discussion sections. We are glad that you found the abstract to be an effective summary of our study and that the methods section was detailed enough to allow for replication. Your acknowledgment of our sample size, recruitment methods, and the robustness of our statistical analyses, feedback on results presentation and discussion session is very encouraging.

 Thank you again for your insightful comments and for appreciating the significance of our contribution to the literature on social media's impact on psychological and behavioral outcomes. We value your feedback and are encouraged by your positive assessment.

 Sincerely,

 Vasudha K.G.

# Reviewer report: Reviewer response for version 1

**DOI:** 10.5256/f1000research.147882.r280599

**Published:** 2024-05-24

**Authors:** Niyaz Panakaje

**Affiliations:** 1 Yenepoya University, Mangalore, Karnataka, India

The study employs a quite interesting approach to assessing the gender differences in body image, exercise motivation, and social comparison among young Instagram users. The present study has a realistic contribution to the existing body of knowledge and feasible recommendations for the young generation. However, some concerns need an immediate attention:

 1.    The introduction of the manuscript is well drafted depicting the background, problem, need, and gap of the study.

 2.    In this section, the authors stated data collection from the sample size of 212 youth, but the rationale behind restricting this number has not been justified which poses a question of generalizability. It is recommended to provide a background for the population and solid justification for collecting only 212 responses (as the study employed convenience sampling, the number of responses needs to be quite high to eliminate the error and bias). The selection of age range is justifiable but the number of sample selections seems to be faulty. Kindly provide a rationale.

 3.    As the study stated “The data was normally distributed with Shapiro-Wilk statistic being 0.845 (>0.05) and mean M = 3.43 and standard deviation SD = 1.50”, the clarification on normality distribution needs to be further explained. As there are three dependent variables, normality needs to be checked for all three variables. However, only one value has been indicated, kindly recheck this part. 

 4.    The study’s implications lack depth. As the study provided highly valuable outcome of disparity in the gender concerning body image, social comparison, and exercise motivation, specific theoretical and practical implications are missing. The paper can benefit by further providing detailed implications to the theories referred and practical scenarios. 

 Overall, the study has the potential to get more visibility due to its novel contributions.

Is the work clearly and accurately presented and does it cite the current literature?

Yes

If applicable, is the statistical analysis and its interpretation appropriate?

Yes

Are all the source data underlying the results available to ensure full reproducibility?

Yes

Is the study design appropriate and is the work technically sound?

Partly

Are the conclusions drawn adequately supported by the results?

Yes

Are sufficient details of methods and analysis provided to allow replication by others?

Partly

Reviewer Expertise:

Higher Education, Stress, Social Media, Entrepreneurhip

I confirm that I have read this submission and believe that I have an appropriate level of expertise to confirm that it is of an acceptable scientific standard, however I have significant reservations, as outlined above.

# Response

**Published:** 2024-06-10

**Authors:** Vasudha K.G

**Affiliations:** Psychiatry, KMC Hospital Attavar, Mangaluru, Karnataka, India

Dear Sir,

 Thank you for your valuable feedback on our manuscript. We appreciate your insightful comments and suggestions, which have undoubtedly contributed to improving the quality of our study.

1. Thank you for acknowledging and giving feedback regarding the introduction of the manuscript.
2. Thank you for your insightful feedback on data collection. We understand the importance of providing a robust rationale for the sample size and addressing concerns about generalizability. We have tried our best to addresses your concerns in the modified manuscript. We've carefully considered your suggestions and believe that the revisions made to the sample size justification adequately address the concerns raised. These modifications aim to provide a clearer rationale for our methodological decisions, ultimately enhancing the rigor of our study. The sample size of 212 participants, though modest, was calculated to achieve a balance between feasibility and statistical power. This size allows for meaningful preliminary insights while acknowledging the need for future studies with larger and more diverse samples to enhance generalizability and validate our findings.
3. We appreciate your observation regarding the need for further clarification on the normality distribution of our data. Upon reviewing our analysis, we acknowledge that normality should be checked for each of the three dependent variables individually. We have provided a detailed explanation and updated our manuscript to include the normality checks for all dependent variables: Body Image, Exercise Motivation, and Social Comparison.
4. We understand the importance of providing detailed theoretical and practical implications based on our findings and we have addressed these points in our revised manuscript. We have added a theoretical implication, like: Self-determination theory and Exercise motivation. We have also mentioned the practical implications, such as complex issues surrounding body image, social comparison, and motivation, particularly in the context of social media and its influence.

# Reviewer report: Reviewer response for version 1

**DOI:** 10.5256/f1000research.147882.r271051

**Published:** 2024-05-06

**Authors:** Vandana Gaur

**Affiliations:** 1 Department of Psychology, SDM Government PG College in Doiwala, Dehradun, Uttarakhand, India

The study effectively explores the relationship between Instagram use and body image, exercise motivation, and social comparison, shedding light on potential sex differences. Methodological rigor and clarity enhance its credibility. However, further analysis on the influence of other variables could enrich the discussion. Overall, a commendable contribution to understanding social media's impact on body perceptions.

Is the work clearly and accurately presented and does it cite the current literature?

Yes

If applicable, is the statistical analysis and its interpretation appropriate?

Yes

Are all the source data underlying the results available to ensure full reproducibility?

Yes

Is the study design appropriate and is the work technically sound?

Yes

Are the conclusions drawn adequately supported by the results?

Yes

Are sufficient details of methods and analysis provided to allow replication by others?

Partly

Reviewer Expertise:

Social Psychology, Cognitive Psychology, Psychopathology

I confirm that I have read this submission and believe that I have an appropriate level of expertise to confirm that it is of an acceptable scientific standard.

# Response

**Published:** 2024-05-07

**Authors:** Vasudha K.G

**Affiliations:** Psychiatry, KMC Hospital Attavar, Mangaluru, Karnataka, India

Dear Reviewer,

 Thank you for taking the time to review our study titled "Sex Difference in Body Image, Exercise Motivation, and Social Comparison among Instagram Users: A Cross-Sectional Study." We appreciate your thoughtful feedback and constructive comments.

 We are pleased to hear that you found our study to effectively explore the relationship between Instagram use and body image, exercise motivation, and social comparison, while also highlighting potential sex differences.

 Regarding your suggestion for further analysis on the influence of other variables, we acknowledge the importance of considering additional factors that may contribute to body image concerns, exercise motivation, and social comparison among Instagram users. In future research, we plan to explore the influence of variables such as self-esteem, media literacy, technology engagement, cultural influences, and peer influences to provide a more comprehensive understanding of these phenomena.

 We are grateful for your positive evaluation of our study's methodological rigor and clarity. We are committed to maintaining these standards in our research endeavors.

 Once again, thank you for your valuable feedback and recognition of our contribution to understanding the impact of social media on body perceptions. We will consider your suggestions for future research directions.

 Regards, 

 Vasudha K.G

 Department of Psychiatry, Kasturba Medical College, Mangalore, Manipal Academy of Higher Education, Manipal, India
